# Host-seeking activity of a Tanzanian population of *Anopheles arabiensis* at an insecticide treated bed net

**DOI:** 10.1186/s12936-017-1909-6

**Published:** 2017-07-04

**Authors:** Josephine E. A. Parker, Natalia C. Angarita Jaimes, Katherine Gleave, Fabian Mashauri, Mayumi Abe, Jackline Martine, Catherine E. Towers, David Towers, Philip J. McCall

**Affiliations:** 1 Department of Vector Biology, Liverpool School of Tropical Medicine, Pembroke Place, Liverpool, L3 5QA UK; 2 Optical Engineering Group, School of Engineering, University of Warwick, Coventry, CV4 7AL UK; 3 National Institute for Medical Research, Mwanza Medical Research Centre, PO Box 1462, Mwanza, Tanzania

**Keywords:** Mosquito, Vector, Behaviour, LLIN, Pyrethroid, Insecticide resistance, Control, Host, Malaria

## Abstract

**Background:**

Understanding how mosquitoes respond to long lasting insecticide treated nets (LLINs) is fundamental to sustaining the effectiveness of this essential control tool. We report on studies with a tracking system to investigate behaviour of wild anophelines at an LLIN, in an experimental hut at a rural site in Mwanza, Tanzania.

**Methods:**

Groups of adult female mosquitoes (n = 10 per replicate) reared from larvae of a local population, identified as predominantly (95%) *Anopheles arabiensis*, were released in the hut. An infrared video tracking system recorded flight and net contact activity over 1 h as the mosquitoes attempted to reach a supine human volunteer within a bed net (either a deltamethrin-treated LLIN or an untreated control net). A range of activities, including flight path, position in relation to the bed net and duration of net contact, were quantified and compared between treatments.

**Results:**

The total time that female *An. arabiensis* spent in flight around LLINs was significantly lower than at untreated nets [F(1,10) = 9.26, *p* = 0.012], primarily due to a substantial reduction in the time mosquitoes spent in persistent ‘bouncing’ flight [F(1,10) = 18.48, *p* = 0.002]. Most activity occurred at the net roof but significantly less so with LLINs (56.8% of total) than untreated nets [85.0%; Χ^2^ (15) = 234.69, *p* < 0.001]. Activity levels at the bed net directly above the host torso were significantly higher with untreated nets (74.2%) than LLINs [38.4%; Χ^2^ (15) = 33.54, *p* = 0.004]. ‘Visiting’ and ‘bouncing’ rates were highest above the volunteer’s chest in untreated nets (39.9 and 50.4%, respectively) and LLINs [29.9 and 42.4%; Χ^2^ (13) = 89.91, *p* < 0.001; Χ^2^ (9) = 45.73, *p* < 0.001]. Highest resting rates were above the torso in untreated nets [77%; Χ^2^ (9) = 63.12, *p* < 0.001], but in LLINs only 33.2% of resting occurred here [Χ^2^ (9) = 27.59, *p* = 0.001], with resting times spread between the short vertical side of the net adjacent to the volunteer’s head (21.8%) and feet (16.2%). Duration of net contact by a single mosquito was estimated at 204–290 s on untreated nets and 46–82 s on LLINs. While latency to net contact was similar in both treatments, the reduction in activity over 60 min was significantly more rapid for LLINs [F(1,10) = 6.81, *p* = 0.026], reiterating an ‘attract and kill’ rather than a repellent mode of action.

**Conclusions:**

The study has demonstrated the potential for detailed investigations of behaviour of wild mosquito populations under field conditions. The results validate the findings of earlier laboratory studies on mosquito activity at LLINs, and reinforce the key role of multiple brief contacts at the net roof as the critical LLIN mode of action.

**Electronic supplementary material:**

The online version of this article (doi:10.1186/s12936-017-1909-6) contains supplementary material, which is available to authorized users.

## Background

The effectiveness of long lasting insecticide-treated nets (LLINs) in malaria prevention [1], and their contribution to reductions in malaria incidence and morbidity in Africa [2], underscore their central role in current and planned malaria prevention and elimination programmes [3–5]. Yet, even as such achievements are being reported, the future of LLINs is under threat from behavioural and physiological changes in mosquito populations; behavioural resistance, such as shifts to feeding outdoors or on animals, could reduce mosquito exposure to LLINs, and the emergence and rapid spread of resistance to the pyrethroid insecticides used on LLINs could decrease the impact of nets [6–8]. Possible solutions to this challenge include using novel insecticides or other treatments delivered via nets, including two-in-one or combination nets [9–12] and novel fabric technologies [13]. Whatever solutions are found to safeguard LLIN effectiveness against pyrethroid-resistant populations, the importance of identifying their mode of action and effect on mosquito behaviour should not be underestimated.

A number of test methods have been used to investigate the behaviour of *Anopheles* sp. when host seeking at LLINs. Baited WHO tunnel tests have been in use for some years to test effects of insecticide on blood-feeding and mortality of approaching mosquitoes [14]. Modified tunnel tests have been adapted for filming, to allow precise quantification of contact with insecticide, and investigation of the host seeking flight of approaching mosquitoes [15–18]. Other approaches have used techniques of tracking and sticky net traps to investigate mosquito activity around human baited LLINs [19–22]. The optical imaging and flight-tracking system used by Parker et al. [21] permitted remote tracking, recording and quantitative analysis of the space around an entire bed net, with multiple mosquitoes simultaneously flying without restriction, over long periods, and responding to the human host ‘bait’ within the bed net. In that report, *Anopheles gambiae *s.s. responses at a human-occupied net were classified into four distinct behavioural modes, termed swooping, visiting, bouncing and resting. Net contact largely comprised multiple brief ‘visits’ or ‘bounces’ on the bed net roof, and the majority of flight paths were above the roof. Behaviour at LLINs was similar to untreated nets inasmuch as insecticide treatment did not repel mosquitoes prior to net contact, although the duration of contact was significantly lower at the LLIN (maximum contact duration in 60 min tests = 96 and 334 s at LLIN and untreated nets, respectively [21]).

The tracking system of Parker et al. [21] was installed in an experimental hut in rural Tanzania and used to describe the flight behaviour and nature of the contact at LLINs for *Culex quinquefasciatus* [23]. Though field tests cannot feasibly control temperature and humidity as is done in laboratory work, a tracking system that produces such high quality information in a natural field setting has many advantages; studies can be carried out on wild rather than colonised populations, minimising the influence of behavioural changes resulting from genetic bottlenecks or unknown selection pressures that might occur during colonisation and the problems associated with laboratory studies of behavioural responses to insecticides can be overcome, e.g. laboratory data are not always consistent with field data [24, 25] and colonisation can result in altered host responses [26, 27] or changes in mosquito responses to insecticides [28–32].

Additional explanations for discrepancies between laboratory and field studies are numerous, but the smaller scale of laboratory test arenas is likely to be a major constraint [33–35], as are the sealed draught-proof laboratory environments that are unlikely to mimic the complex air movements, odour plumes and microclimatic changes that occur in field or more realistic settings [36–39].

The establishment of the field-based tracking system in Tanzania [21] allowed wild mosquito populations to be studied and compared with laboratory studies of colonised *An*. *gambiae *s.s. For this study, we tracked behaviour of *Anopheles arabiensis*, the dominant species in the area. Although they are closely related members within the same species complex and both are primary malaria vectors in Africa, *An*. *gambiae *s.s. and *An*. *arabiensis* differ in a number of key bloodfeeding behaviours. Typically, *An*. *gambiae *s.s. populations are highly anthropophagic, with strong endophagic and endophilic tendencies, while *An. arabiensis* populations feed outdoors as well as indoors and will feed on cattle and other hosts in addition to humans [40–42]. Various studies report species-specific preferences for biting certain areas of the human body, and these preferences may vary according to whether the host is supine, seated or standing [43–45]. This raises the possibility that the arrival site and contact rates of *An*. *gambiae *s.l. species at an LLIN could be influenced by these preferences.

Finally, responses to insecticides may differ between the two species. *Anopheles arabiensis* were reported to be more inhibited by various insecticides during blood-feeding than *An. gambiae *s.s. [46], while another study controlling for insecticide resistance, showed that both species exhibited similar behavioural responses to permethrin in contact irritancy assays [47].

We report here on a study using a large scale tracking system [21] at a rural field site in Mwanza, northern Tanzania to describe and quantify the effects of an LLIN on host-seeking flight of adult female *An*. *arabiensis*. Results were used to validate the results of previous laboratory tests, through qualitative comparison with existing data on behaviour of colonised *An*. *gambiae *s.s. mosquitoes [21].

## Methods

### Mosquitoes

During July and August 2014, anopheline larvae were collected from natural ground water pools in Magu, Tanzania (2°33′41″S 33°18′11″E) and reared to adults in the insectaries at the National Institute of Medical Research in Mwanza. Members of the *An. gambiae *s.l. species complex were identified at adult emergence using morphological keys [40, 48] and maintained under a 12:12 light: dark cycle (approximating the natural cycle) and provided 10% sugar solution ad libitum. Only the first generation of emerged mosquitoes (i.e. F0) were used in the tests.

Initial trials attempted to track wild mosquitoes entering the hut through the eaves or open door after they had emerged naturally from the surrounding area. However, a mixed population of numerous mosquito species within the *Anopheles*, *Culex* and *Mansonia* genera entered the hut, along with numerous other insects from taxa including Diptera, particularly Chironomidae and Chaoboridae, and Lepidoptera. It has not been possible to reliably classify an individual mosquito track as belonging to a specific mosquito genus based on quantified mosquito flight behaviours [23]. Therefore, the approach to use a mixed population of wild mosquitoes was abandoned.

### Species identification and insecticide susceptibility status

The identity of species within the *An*. *gambiae* complex was confirmed periodically throughout the testing period using the standard protocol [49].

In late July (mid-way through the testing period) WHO insecticide susceptibility bioassays were performed on 3–5 day old mosquitoes (n = 22) using 0.05% deltamethrin treated papers, with an exposure time of 1 h following standard methodology [50]. Mortality outcomes at 24 h were corrected according to Abbott’s formula.

### Experimental hut and tracking system

**Fig. 1:**
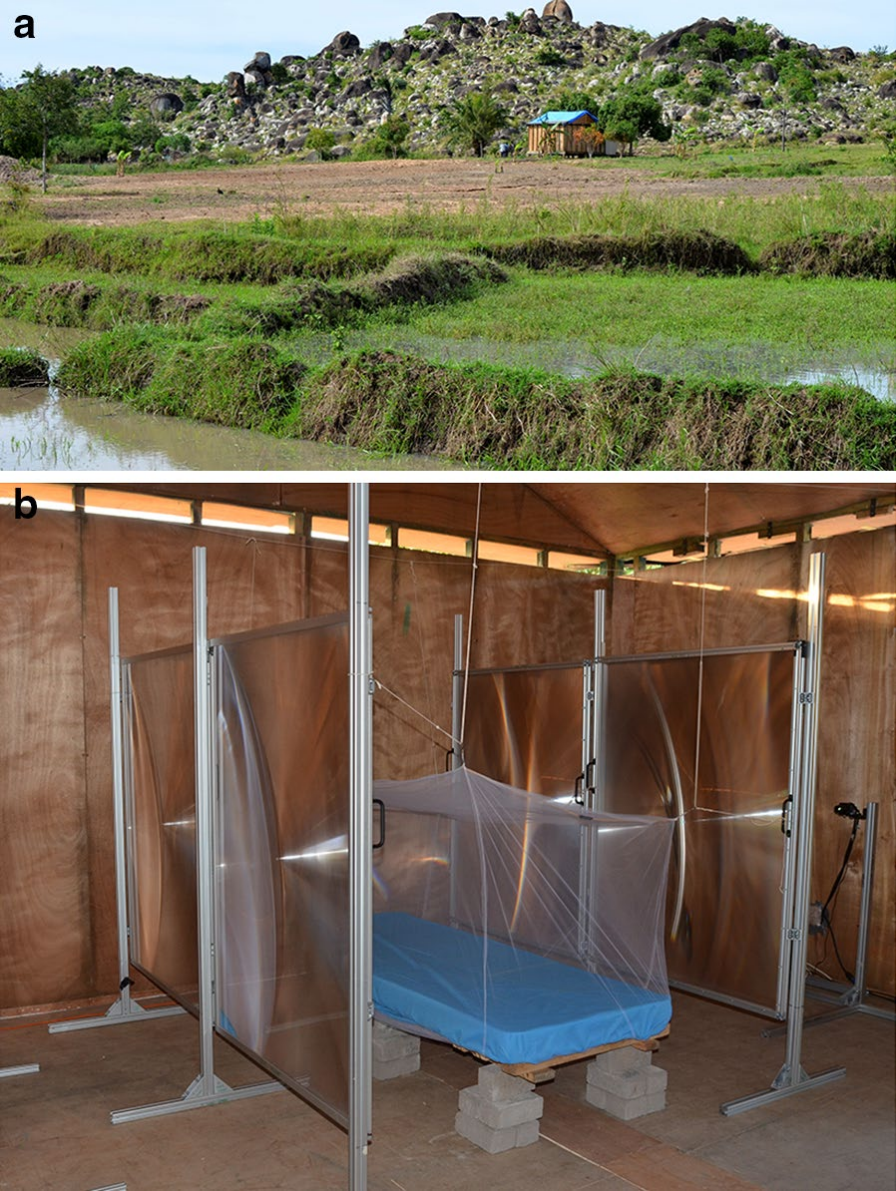
Location and interior of the experimental hut. The* top image* shows the field location of the hut, near Mwanza, Tanzania. The* lower image* shows the bed with the paired Fresnel lenses on either side, with one of the LEDs (mounted on a single aluminium post) visible at the* left edge* of the photograph. The cameras are positioned to the* right*, beyond the frame. Note that in the experiments reported here, the eaves were shut throughout and only mosquitoes reared in the laboratory from wild immature stages were tested

Tests were conducted in a plywood experimental hut, 5 × 5 m floor area (Fig. 1) that had been treated with anti-termite paint more than 12 months prior to use. The floor was supported by six 0.5 m tall timber posts in basins of water with detergent to prevent entry by ants. The walls were 2.5 m high and the roof apex was 3.5 m. The plywood roof panels were covered in plastic tarpaulin and thatch for weather proofing. A 0.1 m eave gap on all four walls was covered with plastic mesh when the reared mosquitoes were released inside the hut to prevent wild mosquitoes entering. Camera cables exited the hut through a port in the wall to a separate small plastic hut where the observer operated the cameras of the tracking system as they recorded events in the experimental hut. Though the system ran automatically the observer was present to operate the active capture process, which was used to avoid recording periods with no mosquito activity [23]. All equipment was powered using a Honda EU20i generator, with a rated output of 1600 W (Seddon Direct, UK). The complete tracking recording system was estimated to draw approximately 600 W, and could be powered by the generator without refuelling for over 6 h.

The experimental hut was erected in a rice growing area near the village of Kayenze (2°23′43″S, 33°0′5″E), approximately 13 km north-east of Mwanza city, Tanzania. The hut was situated 70 m from the nearest house, and 240 m from a cattle enclosure.

### Test procedure

Ten female *An*. *gambiae *s.l. mosquitoes, aged 3–5 days post eclosion, were released in each test. Mosquitoes were selected for experiments 2 h prior to testing by placing an arm against a cage and aspirating those that attempted to feed into a paper cup. Mosquitoes were sugar-starved during transport from the insectary to the field site (typically 1–1.5 h). Tests were conducted between the hours of 21:00 and 00:30, to coincide with peak biting periods of *An*. *gambiae* complex mosquitoes in this region [51–55].

The volunteer entered the bed net 1 h prior to recording, and the paper cup of mosquitoes was hung at eave height on the wall of the experimental hut. At the start of tests, a cord was pulled from outside the experimental hut to remove the net cover from the paper cup, inverting it and releasing mosquitoes into the room. Activity was recorded for 60 min after which the mosquitoes were collected using a prokopack aspirator [56].

Mosquito responses were tracked at Permanet 2.0^®^ LLINs (75 denier polyester net; 55 mg/m^2^ deltamethrin; Vestergaard, Lausanne, Switzerland) and untreated nets (assembled from untreated polyester net of similar mesh), both of which had been tailored slightly to fit the field of view. Ten tests were conducted with each net (i.e. 20 tests in total, using a total of 200 mosquitoes); order of recording mosquitoes at treated and untreated nets was randomised. Up to two tests could be conducted per night, although on days when two tests were conducted the same net was used for both tests.

Members of the local community volunteered to act as human baits; four female and seven male volunteers participated in the ten tests. Half of the volunteers lay with their heads on the left hand side of the field of view, and half with their heads on the right, to control for possible effects of the position of the mosquito release point on net approach.

### Tracking mosquito flights

Mosquito activity was recorded using the tracking system described in detail previously [21, 23]. Two cameras filming at 50 fps recorded a total 1.2 × 2.4 m field of view (1.2 *×* 1.2 m per camera), which was illuminated by two infrared LEDs, with four (two pairs) of large Fresnel lenses (1 × 1.4 m) to collimate the light through the region around the bed net and focus that light into the cameras (Fig. 1b). Each Fresnel lens pair was positioned 1.96 m apart, on either side of the bed. Flight tracking was restricted to the total volume between the two lens pairs combined (Fig. 1b), resulting in a total tracking volume of 1.2 × 2.4 × 1.96 m.

Videos were recorded to sequence (.seq) files using StreamPix software. Mosquito tracks were identified and analysed using custom written Matlab applications [23].

### Statistical analyses

#### Mosquito activity and behavioural modes

Data and observations on flight activity and behavioural modes were obtained and extracted as described previously [21, 23], and summarised in Table 1. Effects of net treatment on activity were analysed in a regression analysis with cluster adjustment of standard errors to account for repeated observations on the same volunteers (STATA). Nine volunteers participated in two tests each (untreated net and LLIN), one volunteer dropped out after the first recording and was replaced by a new individual for the second test. By using cluster adjustment in the regression model, the incorporation of intragroup variation of ‘volunteer identity’ in standard errors was ensured, hence results were less likely to be influenced by variation in attractiveness of any one individual volunteer to mosquitoes.

**Table 1:** Mean total activity time (minutes) of female mosquitoes spent in different behavioural modes over 60 min tests in the field hut

	Swooping	Visiting	Bouncing	Resting	Total
Untreated net	4.0 (2.8–5.7)	11.7 (6.9–19.8)	38.6 (19.5–76.4)	13.4 (7.6–23.5)	73.5 (42.6–126.8)
LLIN	4.9 (3.2–7.5)^–^	8.7 (5.0–15.2)	5.3 (2.5–11.5)*	2.8 (1.7–4.8)	23.8 (14.7–38.5)*

The four behavioural modes are defined as follows. Swooping: tracks that do not contact the bed net. Visiting: tracks where relatively long periods of flight are interspersed with infrequent net contacts; contacts characterized by sharp turns of 80° or more in trajectory; when multiple contacts occur, the minimum interval between them is 0.4. Bouncing: tracks where mosquito makes multiple rapid contacts with the bed net surface at intervals of under 0.4 s; includes short flight events between contacts, or when contact with the bed net surface is maintained but not static. Resting: tracks completely static for at least 0.75 s, or where the speed of mosquito movement is under 1.33 mm/s (equivalent to movement of up to 1 mm in the minimum resting time); constant contact with the bed net surface is assumed

Table shows geometric mean times (minutes) with 95% confidence intervals, from 10 repeat tests per treatment and 10 mosquitoes per test. As multiple mosquitoes were active simultaneously, the total activity time may exceed 60 min

* Indicate results where activity for a given behavioural mode was significantly different between net treatments (p < 0.05). ^–^ indicates that no significant effect of net treatment was found (p < 0.05). Where there is no symbol, testing was not performed (to avoid analysis of multiple non-independent variables, statistical tests were conducted only on swooping and bouncing mode and total activity data)

The effects of net treatment were investigated statistically only for swooping and bouncing behavioural modes, to avoid analysing multiple non-independent variables. As these behavioural categories are not independent, increases in activity in one mode necessarily reduces activity in the others; hence only two categories were selected for analysis: swooping (non-contact flight) and bouncing (flight involving high levels of net contact). Effects of net treatment on time spent in either behavioural mode were assessed using cluster adjusted regression analysis (StataCorp, 2013), as described above. Activity times spent in all behavioural modes are presented in qualitative summaries in graphs, tables and text.

#### Flight speed and tortuosity

Flight speed and tortuosity were calculated as described previously [21] with speed values described only for swooping tracks, because tracks that included net contact were assumed to be slower and would not be equally represented in different net treatments. Tortuosity values were drawn from swooping tracks and the initial sections of net-contacting tracks, up to their point of first contact. Note that since recordings were made in 2D, speed and tortuosity values could potentially underestimate the true values, as track information did not include movement in the z axis. The effect of the explanatory variable of net treatment on the dependent variables of speed and tortuosity was analysed using cluster adjusted regression analysis (StataCorp, 2013), adjusting for repeated tests by the same volunteers.

#### Distribution of activity on the bed net

The field of view was sub-divided into 16 regions (Fig. 4), and activity allocated to different regions for spatial analysis of movement. As recordings were made in 2D, there was a degree of uncertainty over the precise location of mosquitoes within a region. This ambiguity affected whether tracks were assigned to regions 1–10 (the net surface), or regions 15 and 16 (the space surrounding the net). Contact with the net was identified using physical contact definitions detailed below, and used to allocate tracks to the appropriate region.

In spatial analysis of total activity time, and time spent in the four different behavioural modes (Fig. 4b, d–g), time spent in each region was scaled by region size (s/m^2^) to account for the different sizes of the various regions. Physical contact times were analysed by unscaled time (seconds).

Spatial preferences were assessed using a generalised linear model that included terms for net treatment and region, and an interaction term between net treatment and region to assess whether activity was evenly distributed across the field of view (SPSS Statistics, version 21, IBM).

#### Physical contact with the net surface

In this 2D system, mosquito position relative to the net was unknown, and therefore was inferred by examination of track characteristics. Tracks were analysed to find points where mosquitoes made physical contact with the net. Three different track elements were classed as indicating contact with the net; sharp angle turns in tracks (angles of 80° or more [15, 16]); the troughs of bouncing tracks, in which a mosquito changed flight direction at intervals of 0.4 s or less; resting periods, in which a mosquito remained static, or moved at a speed of less than 1.33 mm/s for more than 0.75 s. Examples of all three types of contact can be seen in the Additional file 1: video S1. The total duration of physical contact a mosquito made with the net surface incorporated every contact accrued through all three of these track types.

Effects of net treatment on time spent in physical contact with the net were assessed by cluster adjusted regression analysis (StataCorp, 2013), adjusting for any potential variations resulting from differences in the attractiveness of different volunteers (STATA). As the limits of the camera field of view meant that it was not possible to track individual mosquitoes for the full hour’s test, the range of times that an individual mosquito may have spent in contact with the LLIN was calculated as described in Parker et al. [21]. The maximum of the range makes the assumption that not all mosquitoes released into the room responded to the human bait. The minimum of the range was based on a 100% response rate where all mosquitoes released contacted the net. The maximum of this range was based on a low response rate, calculated by dividing the total contact time by the maximum number of mosquitoes observed responding to the net at one time (i.e. the number of insects we know responded to, and were active around the net). The minimum contact time per individual was calculated by dividing the total duration of physical contact amassed by all mosquitoes by 10 (the total number of mosquitoes released).

#### Activity rates over time

Repellency was quantified using measures of time lag from mosquito release to first appearance in the field of view, and time elapsed until the first mosquito contacted the net. This value was scored for the first mosquito entry only (i.e. one mosquito per test). These values were evaluated using a Log Rank Mantel-Cox survival analysis in SPSS version 21 (IBM). Two tests were conducted to assess the effect of the explanatory variable of net treatment against the outcome variables of time lag to first appearance, and the time between mosquito release and net contact. If nets were repellent, mosquitoes in treated tests would be expected to take longer to appear in the camera field of view, and take longer to make first contact with the net.

Mosquito activity over the entire recording period (60 min) was assessed for suitability for exponential decay modelling, but many of the tests violated that equation’s constraints. Instead, tests were used to assess the difference between activity in the first and the final 5-min intervals (0–5 and 55–60 min). Total activity recorded in the first interval was subtracted from total activity recorded in the final interval: if negative, the value indicated activity decay, while a positive value indicated that activity had increased between mosquito release and termination of the test. These values were compared using cluster adjusted regression analysis (StataCorp, 2013), adjusting for volunteer effects (STATA) to investigate effect of net treatment on attack persistence.

## Results

### Mosquitoes

A total of 142 individuals, morphologically identified as *Anopheles gambiae *s.l., were processed by PCR, and 129 produced a PCR band. Of these, 95.3% (n = 123) were identified as *An*. *arabiensis*, 4.7% (n = 6) as *An*. *gambiae * s.s. Hence, during the study period, adult mosquitoes reared from immature stages from this site were referred to as *An*. *arabiensis.*

Adult female *An*. *gambiae *s.l. from the same population were tested in WHO insecticide bioassays with 0.05% deltamethrin treated papers. Abbott’s correction was applied to 24 h mortality data, as control mortality at this time point was 9.5%. The knockdown rate after 1 h was 95%, the mortality rate after 24 h was 100%, and the population was classified as susceptible to pyrethroids.

### Responses of mosquitoes to LLINs and untreated nets

**Fig. 2:**
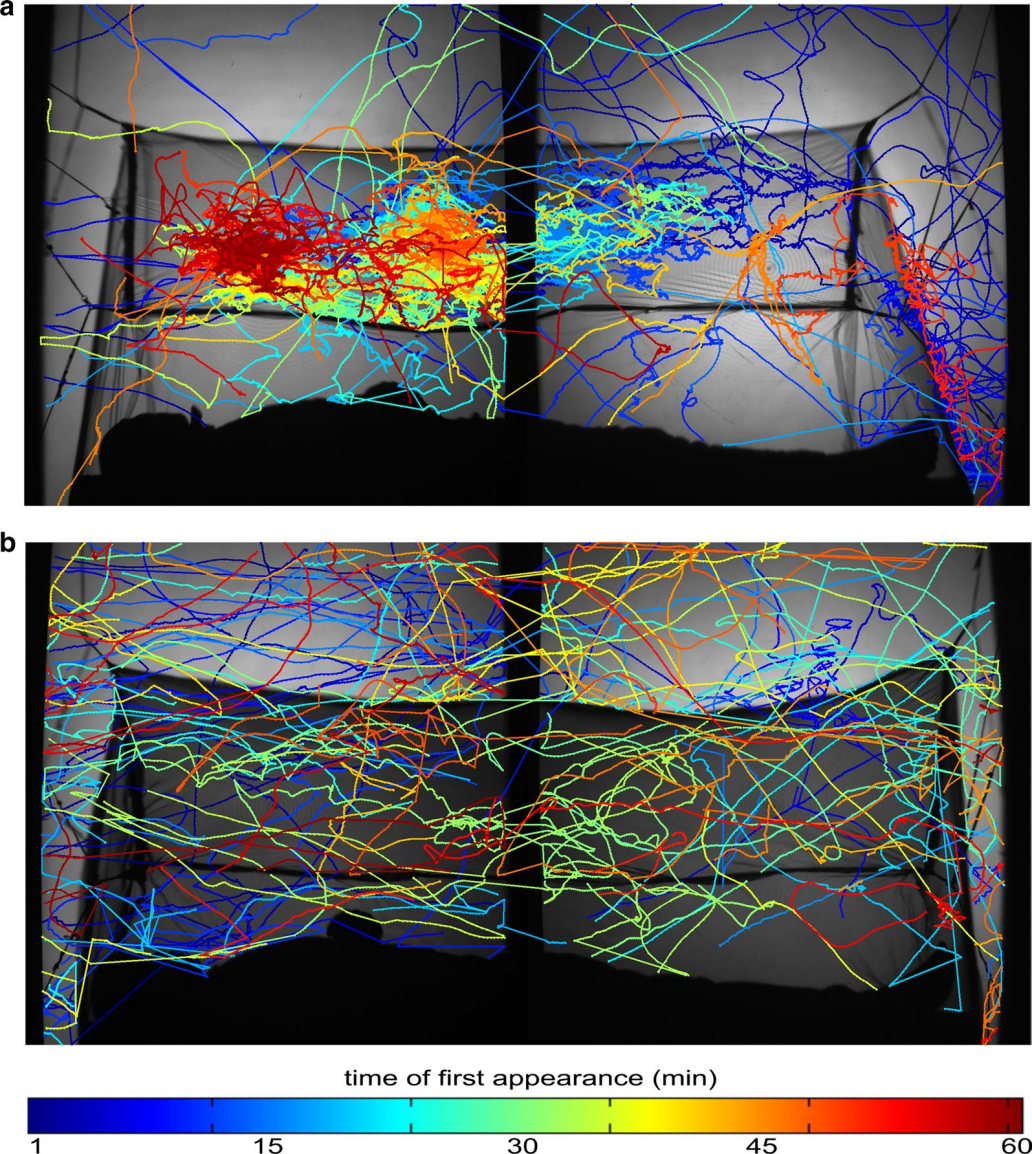
Flight activity of field caught mosquitoes at untreated nets and LLINs. Examples of the flight tracks of *Anopheles arabiensis* in response to a human volunteer inside **a** an untreated and **b** an insecticide-treated bed net (Permanet 2; www.Vestergaard.com). Each image shows the total activity recorded over 60 min, with 10 mosquitoes released in each test. Each track is the path of an individual mosquito flight. Tracks are colour-coded according to the time they first appear in the field of view as shown in the key below the images: *blue* tracks at the start through to *red* at the end of the 60-min test

A total of 20 tests using 200 adult female mosquitoes were completed between 14 July and 23 August 2014. Across both treatments (i.e. 10 untreated net and 10 LLIN test repeats, each using 10 mosquitoes), individual flight track durations ranged from 0.79 s to 13.6 min, with a geometric mean of 1.53 s (95% CI 1.47–1.59; n = 7631 tracks). As Fig. 2 illustrates, the number of flight tracks was greater when the bed net was not insecticide-treated. The total amount of activity per test was significantly higher with untreated nets (73.5 min [95% CI 42.6–126.8]) than with LLINs (23.8 min [95% CI 14.7–38.5]) [F(1,10) = 9.26, *p* = 0.012; difference estimate 62 min, 95% CI 17–109 min].

**Fig. 3:**
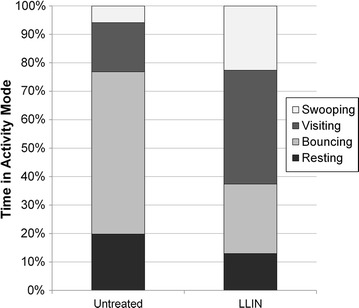
The proportion of time spent by female mosquitoes in each behavioural mode, for the two net types (untreated and LLIN), during tests conducted in the experimental field hut. See caption to Table 1 for definitions of the behavioural modes

Activity distribution between different behavioural modes and analysis of the effects of insecticide treatment on swooping and bouncing modes are shown in Table 1 and Fig. 3. Insecticide treatment did not significantly affect mean times spent swooping [F(1,10) = 1.04, *p* = 0.332], but significantly reduced bouncing activity [F(1,10) = 18.48, *p* = 0.002]. The proportion of the total activity time spent in either bouncing or resting activity, both of which involve contact with the net surface, was 77% at untreated nets and 37% at LLINs (Fig. 3). The proportion of time spent swooping, a mode that is not affected by insecticide treatment, was 6% at untreated nets, and 23% at LLINs, due to the reduction in activity in modes involving net contact (Fig. 3).

A segment of a video recording from the study, showing a sequence of *An. arabiensis* flight around a human-occupied LLIN, and including flights tracks on the roof of the net that exhibit characteristic bouncing and visiting patterns, is provided in the Additional file 1: Video S1 (available online). This clip shows mosquito tracks superimposed over a still reference image of the volunteer baited bednet.

### Flight speed and tortuosity

Swooping mosquitoes in untreated tests flew at a mean instantaneous speed of 327 mm/s (95% CI 306–348). In LLIN tests, the mean swooping speed was 353 mm/s (95% CI 318–388) and was not significantly different from untreated nets [F(1,10) = 3.09, *p* = 0.109].

Track tortuosity was not significantly affected by net treatment [F(1,10) = 0.22, *p* = 0.650]: the mean tortuosity of tracks at an untreated net was 1.35 (95% CI 1.20–1.50), and 1.40 (95% CI 1.27–1.53) at LLINs.

### Location of activity at the bed net interface

Spatial analysis was conducted to investigate effect of region, insecticide treatment, and potential interaction between region and insecticide on the outcome activity density (i.e. activity scaled by region size). In these models, insecticide treatment significantly reduced total activity levels [Χ^2^ (1) = 17.81, *p* < 0.001]. This decrease was seen in bouncing [Χ^2^ (1) = 16.01, *p* < 0.001] and resting [Χ^2^ (1) = 21.96, *p* < 0.001] modes, but not swooping [Χ^2^ (1) = 3.77, *p* = 0.052] or visiting [Χ^2^ (1) = 0.92, *p* = 0.337].

**Fig. 4:**
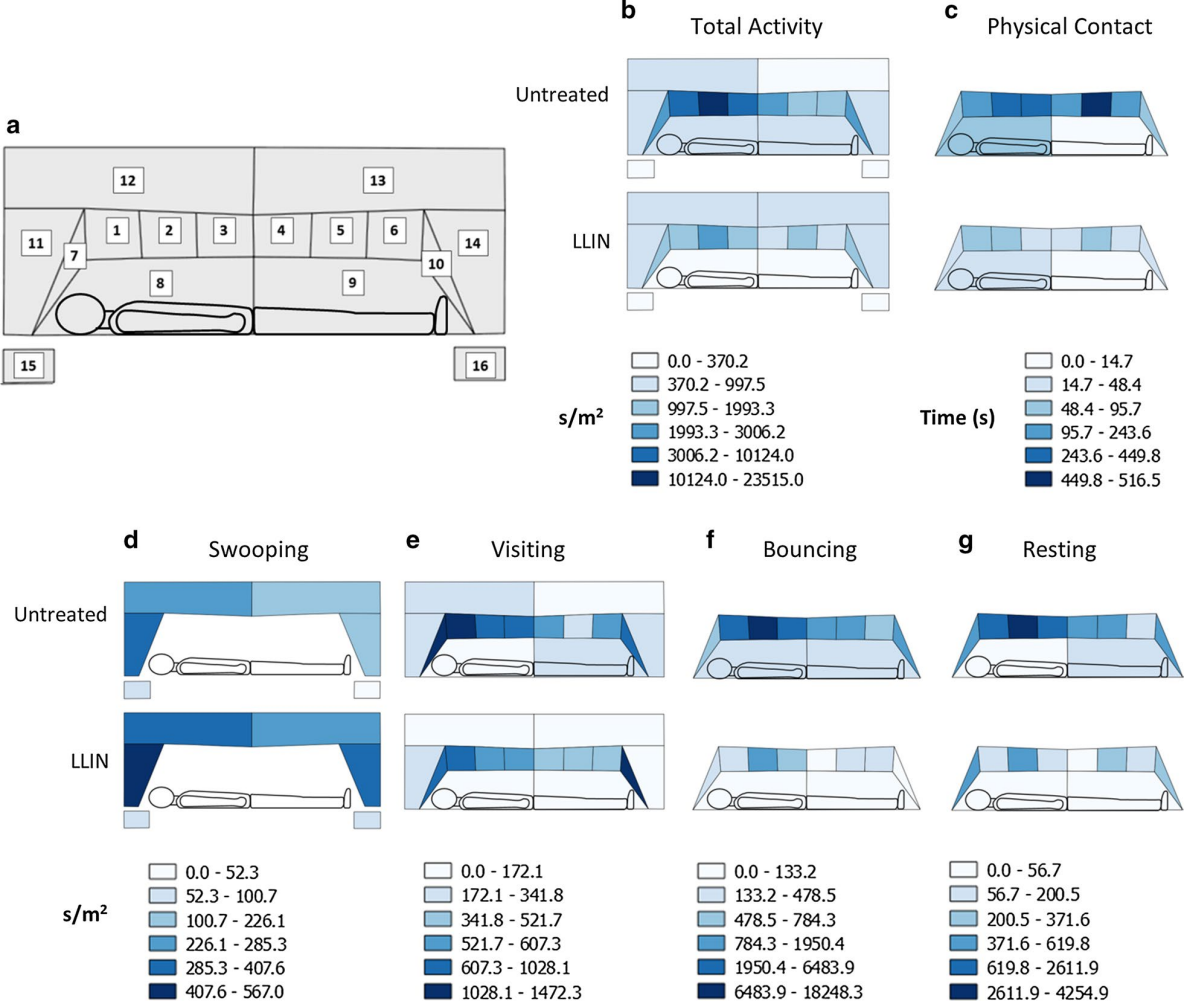
Distribution maps of *Anopheles arabiensis* flight activity on and around untreated and treated bed nets. **a** Distribution map key showing region codes for different areas of the field of view. Regions 1–6 represent the surface of the bed net roof; 7 and 10 are the vertical surfaces at the head and foot ends; 8 and 9 are vertical side surfaces. Flight activity in the space around the net was assigned to regions 11–14. Regions 15 and 16 contain swooping activity (i.e. flight without net contact) occurring in front of the net, on the* left* (15) and* right* (16) sides of the field of view. **b** Density of total activity, i.e. in all behavioural modes (s/m^2^). **c** Distribution of physical contact with the net (in seconds), including resting mode, and brief mid-flight contact made during flight trajectories in the visiting and bouncing modes. (**d**–**g**) Distribution of activity for each behavioural mode—swooping (**d**), visiting (**e**), bouncing (**f**), resting (**g**)—with values expressed as activity density (s/m^2^). Colour coding is specific to each image, as shown in the legend beneath each chart. Charts only include regions relevant to each behavioural category, hence the swooping chart (**d**) does not use net regions 1–10, and the resting chart (**g**) does not include the space around the net (regions 11–16). Although tests controlled for the orientation of the human bait in relation to the mosquito release point, all figures show the volunteer with the head on the left

Accounting for these effects, generalised linear models also indicated spatial differences in activity distribution (Fig. 4). Total activity density was unevenly distributed across the entire field of view [Χ^2^ (15) = 234.69, *p* < 0.001], with most activity occurring on the net surfaces (regions 1–10). Only 3.8 and 15.9% of total activity occurred in the spatial regions around the bed net in untreated net and LLIN tests respectively. The majority of activity occurred on the net roof (regions 1–6: 85.0% on untreated nets, 56.8% on LLINs; Fig. 4b), with a lesser proportion occurring on the vertical net end next to the volunteer’s feet (region 10, 4.6% untreated, 2.0% LLIN). There was a significant interaction between net treatment and total activity distribution [Χ^2^ (15) = 33.54, *p* = 0.004]: the proportion of activity occurring in regions 1–3 (i.e. over the host torso) was significantly higher for untreated nets (74.2%) than for LLINs (38.4%).

Only 10.4 and 10.9% of all swooping at untreated nets and LLINs respectively occurred in regions 15 and 16 (the spaces in front of the bed net, see Fig. 4a), the lowest levels of this behaviour mode in any region [Χ^2^ (5) = 66.77, *p* < 0.001]. Net treatment did not affect distribution of swooping flights [Χ^2^ (5) = 3.71, *p* = 0.592; Fig. 4d].

Most visiting activity occurred on the roof of the net above the volunteer’s torso regions 1–3 [39.9% on untreated nets; 29.9% on LLINs; Χ^2^ (13) = 89.91, *p* < 0.001]. Regions 7 and 10 accounted for 16.1 and 10.2% respectively of visiting activity on untreated nets, and 13.7 and 20.8% at LLINs. Net treatment did not significantly affect the distribution of visiting behaviour [Χ^2^ (13) = 10.42, *p* = 0.659; Fig. 4e].

The majority of bouncing activity occurred in region 2 on both untreated nets (50.4%) and LLINs [42.4%; Χ^2^ (9) = 45.73, *p* < 0.001; Fig. 4f]. In contrast, very low levels of bouncing occurred at the lower body portion of the net regions 4–10 (16.1% of untreated bouncing, 31.5% of LLIN bouncing occurred in these seven regions). Net treatment affected distribution of bouncing [Χ^2^ (9) = 28.14, *p* = 0.001], with significantly higher bouncing rates at region 2 of the roof of untreated nets (Fig. 4f).

Activity in resting mode was unevenly distributed between net regions [Χ^2^ (9) = 63.12, *p* < 0.001]. High levels of resting were observed on region 2 above the volunteer’s chest in both untreated nets (38.6%) and LLINs (21.0%; Fig. 4g). However, there were significant differences [Χ^2^ (9) = 27.59, *p* = 0.001] in distribution of resting events according to net treatment: in LLINs, the highest density of resting (21.8%) was recorded on the vertical surface of the net adjacent to the head (region 7), where only 5.6% of resting occurred on untreated nets. Thus, the majority of resting events occurred on regions 1–3 (77% of total) at untreated nets, but at the LLIN only 33.2% of resting occurred here (Fig. 4g).

### Quantifying duration of net contact

Since the levels of physical contact with the net are calculated from the combination of visiting, bouncing and resting activity, the distribution and duration of net contact mirrors the relative preferences for those behavioural modes (Fig. 4c). Hence, the highest level of physical contact occurred in region 2 on both net types, where the mean total duration (by ten mosquitoes) of net contact per test was 774 s in untreated nets and 126 s in LLINs [equivalent to 37.7 and 26.6% of total contact time; Χ^2^ (9) = 30.09, *p* < 0.001]. The frequencies of net contact at different regions were influenced by net treatment [Χ^2^ (1) = 20.00, *p* = 0.011]: in untreated nets, the majority of net contact (76.7%) occurred at roof regions 1–3, whereas in LLINs, net contact occurred at 1–3 (47.0%) and at roof regions 5 and 6, above the volunteer’s feet (21.2%).

Total net contact duration was significantly higher in untreated nets than LLINs [Table 2; F(1,10) = 10.07, *p* = 0.010 (mean difference = 1572 s; 95% CI = 468–2675)]. The longest contact time recorded for a single track was 285 s on an untreated net, and 155 s on an LLIN. Since multiple mosquitoes were present in all tests and it was not possible to follow an individual mosquito once it had left the field of view, determining the total number of mosquitoes responding per test or tracking individual mosquitoes throughout a test was not feasible. These tracking limitations meant that we were unable to measure the actual total contact time for an individual mosquito during the 60 min, and information on the maximum number of mosquitoes observed simultaneously active in the field of view was used to calculate a range of plausible estimates of contact time for single mosquitoes, as described in the caption to Table 2. On this basis, the ranges of net contact duration were estimated at 204–290 s at an untreated net, 46–82 s at an LLIN over the 60-min test.

**Table 2:** Duration of *Anopheles arabiensis* physical contact with a bed net, during a 60-min test

Duration of physical contact with the bed net surface (60 min test)			
	Mean total time (all contacts)^a^ (min)	Mean time/mosquito (10 mosquitoes)^b^ (s)	Mean time/mosquito (observed max number)^c^ (s)
Untreated net	33.9 (15.78–52.1)	204	290
LLIN	7.75 (4.43–11.05)	46	82

Table shows net contact duration as calculated for ^a^ the mean total time of all contacts observed (all mosquitoes); ^b^ the minimum mean contact time per mosquito, assuming all 10 mosquitoes responded, and ^c^ the calculated maximum mean contact time per mosquito, based on the maximum number of individual mosquitoes observed simultaneously at any time in each test. Values shown are means with 95% CI

Mean total contact time was significantly higher at untreated nets than at LLINs (p = 0.010)

### Responses to the bed net over time

The results did not provide any evidence for any repellent effect of the LLIN prior to net contact. The delay prior to the first mosquito’s appearance in the field of view was not significantly affected by net treatment [Χ^2^ (1) = 0.60, *p* = 0.438], with a geometric mean delay from release to first appearance of 8 s (95% CI 4–14) in untreated nets, and 16 s (95% CI 1–39) in LLINs.

In untreated nets, mosquitoes first contacted the net at a geometric mean of 36 s (95% CI 7–89) after release, compared to 46 s (95% CI 9–119) in LLINs, times that were not significantly different [Χ^2^ (1) = 0.89, *p* = 0.766].

**Fig. 5:**
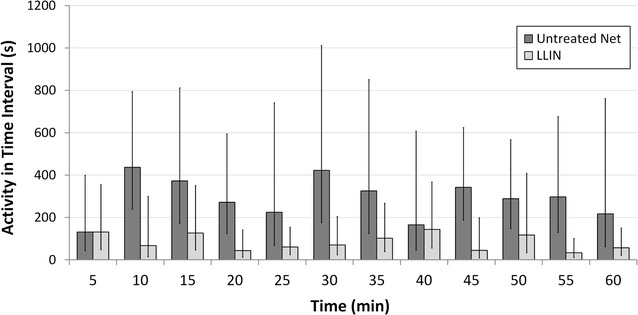
Rates of *Anopheles arabiensis* activity at a human-occupied bed net throughout the 60-min test period. Values show geometric mean (±95% CI) per 5-min interval of the 60-min test. i.e. 5 (0–4 min 59 s), 10 (5 min–9 min 59 s) etc.

Individual test results could not be modelled for exponential decay over the 60-min test, as only 5 of 10 untreated net tests and 8 of 10 LLIN tests fitted model assumptions of decreasing activity over time. As shown in Fig. 5, mosquito activity in untreated net and LLIN tests commenced at similar levels but showed different trends over time, with a greater decrease in activity seen with LLINs [F(1, 10) = 6.81, *p* = 0.026].

## Discussion

The study investigated the behaviour of a wild *Anopheles* females responding to a human bait within an insecticide-treated bed net, in the field in Africa. The mosquitoes were a pyrethroid susceptible population comprising predominantly *An*. *arabiensis* and the results show detailed flight data on host seeking by wild mosquitoes under natural conditions.

Following widespread use of IRS and LLINs, which target endophily and endophagy respectively, *An*. *arabiensis* is becoming increasingly important as a vector of residual malaria in Africa [53, 57], a consequence of its high levels of zoophilic, exophagic and exophilic behaviours. Nonetheless, some levels of endophilic activity still occur as evidence indicates that LLINs are still effective against *An*. *arabiensis* [58–60].

Comparing responses at LLINs with untreated control nets, insecticide treatment reduced net attack (as measured by the number or frequency of flights), chiefly affecting bouncing and resting, the behavioural modes involving highest levels of net contact. The majority of flight activity occurred on the bed net roof in the area above the volunteer’s torso (regions 1–3; Fig. 4b), though this preference was more pronounced in untreated nets than LLINs. Net contact estimates indicated that an individual mosquito accumulated at least 46 s of physical contact with the LLIN during a 60 min test. Since no evidence was found for insecticide repellency, the LLIN appeared to exert its effect only after direct contact with the insecticide, resulting in reduced activity.

These findings are broadly similar to those recently reported using the same tracking system and a long-established colony of *An*. *gambiae *s.s. under laboratory conditions [21]. Considering that fewer *An. arabiensis* were released in this field study (10 mosquitoes per test) than in the laboratory (25 *An*. *gambiae *s.s. per test), activity per mosquito was higher in the *An. arabiensis* field population: total activity time at untreated tests was 91.9 min for *An*. *arabiensis*, compared to 124.6 min for *An. gambiae *s.s. Insecticide treatment reduced activity to 32% of untreated net values with *An. arabiensis* in the field, compared to a reduction to 17% with *An*. *gambiae *s.s. Whether these differences resulted from colonisation, innate differences between these species, or differences in test conditions and setting, is impossible to determine. However, earlier work has suggested that some mosquito species are more active than others during host-seeking and in behavioural bioassays [20, 23, 61, 62].

As seen in previous laboratory reports with other *Anopheles* spp., total activity, bouncing, and resting flight densities were highest at the net roof above the volunteer’s torso in all treatments, although insecticide treatment significantly lowered this preference relative to other areas of the net (see track examples in Additional file 1: video S1) [20–22]. Notably, in both the present study and the earlier *An*. *gambiae *s.s. study [21], the proportions of activity on the roof (Fig. 4; regions 1–6) and foot end of untreated nets (region 10) were comparable: *An. arabiensis,* 85.0 and 4.6%, *An*. *gambiae *s.s., 74.7 and 10.9% at roof and foot regions, respectively. At LLINs however, the equivalent values were markedly different between the two studies with roof and foot end activity at 56.8 and 2.0% in the *An. arabiensis* field study, and 78.3 and 8.8% in the *An*. *gambiae *s.s. laboratory study, respectively. Nevertheless, the majority of mosquito activity was focused on the roof of a human-occupied bed net, both here and in the earlier study [63].

Little activity was observed at the sides of the net, an important observation as this is the region where nets are most likely to be damaged, particularly so at the bottom of the net close to the mattress, where holes are most commonly found [64]. We acknowledge that the 2D nature of our tracking system could have underestimated net contacts in regions 8 and 9, as the sharp angled movements towards and away from the net that comprise ‘visiting’ might not have been visible in every case and some events potentially could have been misclassified as ‘swooping’. However, since total activity (Fig. 4b), which includes swooping, upholds the mosquito preference for the net roof observed when measuring total physical contact times (Fig. 4c), misclassifications would not have been significant. Moreover, sticky net studies, which trap mosquitoes that touch the net, also showed similar attacking patterns [20, 22].

The use of 10 mosquitoes per test, compared with 25 in previous laboratory studies, provided a more precise estimate of net contact times per mosquito. In laboratory tests, a single *An*. *gambiae *s.s. accumulated 18–96 s of net contact in a 60 min test [15], whereas in the field tests with *An. arabiensis,* the range was 46–82 s. However, in the present study, mortality was not recorded as it was not possible to recapture all mosquitoes in the experimental hut following tests, and those that were collected were often physically damaged by the prokopak aspirator, resulting in elevated mortality rates. Elsewhere, in flight tracking experiments that precisely quantified LLIN contact, *An. gambiae* that contacted deltamethrin for 40 s or more were knocked down at 1 h, and dead after 24 h, although the number of mosquitoes tested was low (6/35 tested were knocked down) [16]. The insecticide concentration used was comparable with that in the present study and as susceptible *An*. *arabiensis* and *An*. *gambiae *s.s. display similar knock-down times [47], it is reasonable to assume that mosquitoes in our study would have been affected 1–24 h after net attack.

Here, and in a previous study [15], the nature and duration of insecticide exposure at an LLIN have been revealed as being markedly lower than that used in the standard bioassays for assessing KD_50_ [65], where mosquitoes are forced into almost continuous contact with insecticides for a constant 3 min. The validity of such tests should be re-evaluated, while investigating the consequence for mosquitoes of more realistic or natural types of exposure should be prioritised, aiming for more accurate measurement of net effectiveness and mosquito susceptibility.

Decay in mosquito activity over the hour’s test was studied to assess whether mosquitoes desisted attacking LLINs following initial contact with insecticide. There was little evidence for such striking temporal decay in activity by *An*. *arabiensis* at LLINs as had been reported in laboratory tests with *An*. *gambiae *s.s., where high initial activity fell rapidly to negligible levels within 30 min of release [21]. Though less obvious here, where activity of *An*. *arabiensis* at LLINs remained at relatively low levels throughout, the reduction in activity over the 60 min of testing was greater at LLINs than with untreated nets (Fig. 5). As previously stated, whether this is the result of differences between a relatively homogeneous colonised strain and a heterogeneous wild population, differences in experimental conditions or between the two species, remains to be determined. No volatile or pre-contact repellent effects of the LLINs had been observed in either study, suggesting that observed behaviours were responses by mosquitoes after LLIN contact. This is supported by a recent report that LLINs did not affect house entry rates [66]. A limitation of our study was that the closed test room design prevented the detection of exiting behaviour following net contact. Clearly, further work on the insecticides used on bed nets is needed to characterise the range of possible effects (e.g. toxic, irritant, sensory impairment) and their impact on mosquitoes (e.g. immediate/delayed, reversible, lethal, consequences post-blood meal, etc.), many of which might be subtler than previously recognised.

Neither this study nor the earlier laboratory study [15] detected any effects of net treatment on track speed and tortuosity. Speeds of *An. arabiensis* in all baited tests were between 322 and 355 mm/s, and tortuosity ranged from 1.36 to 1.66. In the laboratory, *An*. *gambiae *s.s. speeds were comparable [21]: mean speeds ranged from 321 to 327 mm/s, and mean tortuosity values were between 1.63 and 1.66. Tracking in 2D will lead to underestimation of true speeds as the system does not record movement in the z axis. Nonetheless, flight speeds recorded in the present study with *An*. *arabiensis* were comparable to those observed in 3D tracked wind-tunnel host seeking experiments, where upwind velocities ranged from 50 to 444 mm/s, and the highest average downwind flight velocity recorded was 272 mm/s [67, 68].

Conditions in the field hut used in the present study differed to those of the previous laboratory study [15] in a number of ways. Field tests were conducted in a slightly larger room with a higher ceiling and some air movement, with wild mosquitoes, at night time and with naturally fluctuating temperature, humidity and lunar illumination. However the similarity between these two studies, and indeed a third report using the tracking system [16], indicate that data obtained in the laboratory can be considered a reliable representation of LLIN performance in a natural context. On this basis, the key role of the bed net roof for targeting *Anopheles* sp. and *Culex* sp. and delivering insecticide via multiple brief contacts, is reinforced by the results reported here.

## Conclusions

The study has demonstrated the potential for detailed investigations of mosquito behaviour under semi-field conditions. The results validate the findings of earlier laboratory studies on mosquito activity at LLINs, and reinforce the evidence for ‘attract and kill’ via multiple brief contacts at the net roof as the principal mode of action of LLINs.

## Additional file

**Additional file 1: Video S1.** Mosquito flight at a human-occupied LLIN in an experimental hut in Mwanza, Tanzania, July/August 2014. The video is a 45 s segment from a recording of activity of *Anopheles arabiensis* at a human-occupied Permanet 2 LLIN in the experimental hut in the field. The flight tracks on the roof of the net exhibit bouncing and visiting behaviour, before one mosquito departs from the net and exits the field of view. The clip shows mosquito flight tracks superimposed on a still reference image of the volunteer baited LLIN. Video available at: https://www.dropbox.com/s/n2l4d8eqd92pcyv/SupplementaryVideo_Chapter6_1.avi?dl=0.
